# Identification of components in Kazakhstan honeys that correlate with antimicrobial activity against wound and skin infecting microorganisms

**DOI:** 10.1186/s12906-021-03466-0

**Published:** 2021-12-20

**Authors:** Pauline McLoone, Aizhan Zhumbayeva, Sofiya Yunussova, Yerkhat Kaliyev, Ludmila Yevstafeva, Susan Verrall, Julie Sungurtas, Ceri Austin, J. Will Allwood, Gordon J. McDougall

**Affiliations:** 1 Department of Biomedical Sciences, School of Medicine, Nazarbayev University, Kabanbay Batyr 53, Nur-Sultan, Kazakhstan 0100000; 2 Medical Microbiology, Republican Diagnostic Center, University Medical Center, Nur-Sultan, Kazakhstan; 3 Information and Computational Sciences Department, The James Hutton Institute, Invergowrie, Dundee, Scotland, UK; 4 Plant Biochemistry and Food Quality Group, Environmental and Biochemical Sciences Department, The James Hutton Institute, Invergowrie, Dundee, Scotland, UK

**Keywords:** Antimicrobial activity, Kazakhstan honeys, Liquid chromatography with mass spectrometry, Polyphenols, Wound infections, Skin disease

## Abstract

**Background:**

Antimicrobial drug resistance is a major public health threat that can render infections including wound and skin infections untreatable. The discovery of new antimicrobials is critical. Approaches to discover novel antimicrobial therapies have included investigating the antimicrobial activity of natural sources such as honey. In this study, the anti-microbial activity and chemical composition of 12 honeys from Kazakhstan and medical grade manuka honey were investigated.

**Methods:**

Agar well diffusion and broth culture assays were used to determine anti-microbial activity against a range of skin and wound infecting micro-organisms. Folin-Ciocalteu method was used to determine the total phenol content of the honeys and non-targeted liquid chromatography analysis was performed to identify components that correlated with antimicrobial activity.

**Results:**

In the well diffusion assay, the most susceptible micro-organisms were a clinical isolate of Methicillin resistant *Staphylococcus aureus* (MRSA) and *Enterococcus faecalis* (ATCC 19433). Buckwheat & multi-floral honey from Kazakhstan demonstrated the highest antimicrobial activity against these two micro-organisms. Kazakhstan honeys with a buckwheat floral source, and manuka honey had the highest total phenol content. Non-targeted liquid chromatography analysis identified components that correlated with anti-microbial activity as hydroxyphenyl acetic acid, *p*-coumaric acid, (1H)–quinolinone, and abscisic acid.

**Conclusions:**

The Kazakhstan honeys selected in this study demonstrated antimicrobial activity against wound and skin infecting micro-organisms. Compounds identified as correlating with antimicrobial activity could be considered as potential bioactive agents for the treatment of wound and skin infections.

**Supplementary Information:**

The online version contains supplementary material available at 10.1186/s12906-021-03466-0.

## Background

Anti-microbial drug resistance is a major worldwide health problem requiring the urgent discovery of novel therapeutic interventions [1]. The treatment of wound and skin infections with antibiotics, for example, has become less effective because of infection with antibiotic resistant strains of bacteria such as Methicillin resistant *Staphylococcus aureus* (MRSA) [2]. Manuka honey, produced by bees (*Apis mellifera*) gathering nectar from the flowering plant *Leptospermum scoparium*, has broad range anti-bacterial activity and is recommended for the clinical management of wound infections [3]. Common wound infecting micro-organisms include *Staphylococcus aureus*, *Pseudomonas aeruginosa*, *Escherichia coli*, *Enterococcus faecalis* and *Acinetobacter baumannii* [2]. In addition to wound infections, micro-organisms are associated with the etiology of a variety of skin disorders. For example, *S. aureus* is a cause of impetigo and furuncles and skin colonization with *S. aureus* is a common feature of atopic dermatitis [4]. Furthermore, *Candida albicans* is a cause of cutaneous candidiasis and Malassezia yeasts have been associated with seborrheic dermatitis and tinea versicolor [5]. As honey has been shown to have broad range anti-microbial activity, it is realistic to consider honey as a potential therapeutic agent for other skin disorders where micro-organisms are involved in the mechanisms of the disease.

Research has shown that components responsible for the anti-microbial activity of honey include high sugar content, low pH, hydrogen peroxide (H_2_O_2_), antimicrobial peptides, methylglyoxal (MGO) and polyphenols [6]. All honeys are unique, and the chemical composition and anti-microbial activity is often variable between different types of honey. The low moisture and high sugar content of honey exerts osmotic pressure on bacterial cells whilst low pH denatures bacterial proteins and inhibits growth. Interestingly, there is recent interest in sugar as a treatment for wounds, as well as osmotic pressure, sugar is thought to reduce amino acid breakdown in the wound leading to a reduction in wound odor [7]. Honey also contains a mixture of polyphenols derived from plant nectar, which have been described as giving each honey a polyphenol signature [8]. Polyphenols have been shown to have bactericidal effects, for example, the phenolic compound methyl syringate has been identified in manuka honey and reportedly has anti-microbial activity [9]. Polyphenols may act alone or synergistically with other polyphenols or other antimicrobial components in honey to kill micro-organisms [10]. The identification of polyphenols in honey that correlate with antimicrobial activity could help in the discovery of new antimicrobial compounds or bioactive agents of dermatological importance.

Of all the honey types, manuka has been the most extensively investigated, however, there may be others with superior antimicrobial activity that are still to be recognized. Honeys of a variety of floral sources are produced abundantly across Kazakhstan, yet the regional honeys have not been fully investigated for biological activity and potential therapeutic use. Hence, the primary aim of this study was to investigate the antimicrobial activity of selected honeys from Kazakhstan against wound and skin infecting micro-organisms and compare their activity with medical grade manuka honey. Secondly, we investigated the composition of the honeys, including pH, sugar content and total phenol content (TPC) and carried out non-targeted liquid chromatographic mass spectrometry (LC-MS) to analyze the metabolic profiles of the honeys, with the objective of identifying components responsible for the anti-microbial activity.

## Methods

### Microbial strains

For the well diffusion assay, the following clinical isolates obtained from the Medical Microbiology Department, Republican Diagnostic Center (RDC), Nur-Sultan, Kazakhstan were used: Methicillin resistant *Staphylococcus aureus* (MRSA), *Acinetobacter baumannii* (multi-drug resistant), *Klebsiella pneumoniae* (multi-drug resistant) and *Candida albicans* (sensitive*)* (Anonymous patient data showing resistance/sensitivity of the clinical isolates to a range of antibiotics or antifungals is provided in Supplementary file 1). Identification of the clinical isolates was performed using VITEK-MS: Healthcare spectrophotometer apparatus. The cefoxitin disc diffusion assay was used to confirm that the MRSA strain was methicillin resistant [11]. Other microorganisms were from the American Type Culture Collection (ATCC) and included: *Pseudomonas aeruginosa* (ATCC 27853), *Staphylococcus aureus* (ATCC 29213), *Escherichia coli* (ATCC 25922), *Enterococcus faecalis* (ATCC 19433) and *Malassezia furfur* (ATCC 14521)*.* For the broth culture assay, *Staphylococcus aureus* (ATCC 25923), *Pseudomonas aeruginosa* (ATCC 27853) and *Escherichia coli* (ATCC 25922) were used. Results of antibiotic susceptibility testing for these three strains are shown in Tables S1a-c (Supplementary file 2).

### Honey samples

The following Kazakhstan honeys (numbered 1–12) were analysed: 1. Multi-floral (East Kazakhstan*); 2. Sweet Clover (Akmola region**); 3. Sunflower (Akmola); 4. Multi-floral (East Kazakhstan); 5. Buckwheat (East Kazakhstan); 6. Buckwheat (Burabay); 7. Buckwheat & multi-floral (East Kazakhstan); 8. Buckwheat (2nd batch of honey 5); 9. Multifloral (2nd batch of honey 1); 10. Sweet clover (2nd batch of honey 2); 11. Sunflower (2nd batch of honey 3); 12. Multi-floral (2nd batch of honey 4). *Borodulikha village, towns of Zyryanovsk and Shemonaikha **Shortandy and Alexeyevka villages. All the Kazakhstan honeys were supplied by apiarists based in Nur-Sultan, Kazakhstan except for honey 6 which was purchased from a local market. The apiarists use beehive trailers to reach different regions of Kazakhstan. Activon 100% medical grade manuka honey (Advancis Medical, UK) was also tested. To determine the effects of sugar on bacterial growth, a sugar control was also produced and was based on the sugar composition of honey (consisting of 167.5 g glucose, 202.5 g fructose, 37.5 g maltose, 7.5 g sucrose dissolved in 85 mL of sterile distilled water) [12].

### Determination of background microbial contamination of honey

Samples of each honey were spread onto plates containing Mueller Hinton agar (Sigma Aldrich) and incubated aerobically at 37 °C. After 24 h, plates were examined for microbial contamination.

### Determination of the antimicrobial activity of honeys in vitro using a well diffusion assay

An agar well diffusion assay [13] with some modifications was adopted to determine the susceptibility of the micro-organisms to the different types of honey. Briefly, sterile glass petri dishes, 100 mm in diameter, were filled with 25 mL of nutrient agar (Himedia, India) that had been inoculated with the relevant micro-organism at a turbidity of 0.5 McFarland standard. Sabouraud dextrose agar (Himedia) was used for *C. albicans* and *M*. *furfur*. Using a sterile cork borer, wells of 8 mm in diameter and 3.2 mm height were cut in the agar. Samples of Kazakhstan honeys, manuka honey and sugar solutions were added to the wells in equal volumes at concentrations of 100, 75 and 50% (w/v) diluted in sterile distilled water. These concentrations were selected because of their clinical relevance (for wound healing, honey is commonly applied topically at concentrations between 90 and 100%). For the 100% honey concentrations wells were filled to the surface and for the 75 and 50% concentrations 100 μl volumes were added. Zones of inhibition were measured in mm with a clear ruler 24 h after aerobic incubation in a 37 °C incubator. All experiments were conducted in either duplicate or triplicate and repeated two, three or four times on separate occasions.

### Determination of the antimicrobial activity of honeys in vitro using a broth culture assay

A broth culture assay was used to determine the antibacterial activity of the Kazakhstan honeys (7–12) and the manuka honey against *S. aureus*, *P. aeruginosa* and *E. coli* using the method of Schneider et al. [14]. Briefly, 75% honey broth cultures were prepared in tryptone soya broth (TSB) (Sigma Aldrich) (7.5 g of honey made up to 10 mL with TSB) then inoculated with 100 μl of an overnight culture of the micro-organism and incubated for 24 h aerobically at 37 °C at 120 rpm. Samples (1 mL) of the broths were serially diluted in 0.1 M sterile Phosphate Buffered Saline (PBS). The neat broth and serial dilutions (100 μL) were spread onto tryptone soya agar plates using a sterile spreader. Plates were placed in a 37 °C incubator for 24 h in aerobic conditions. 100% TSB control and 75% of the sugar control were also tested. Plates that had between 30 and 300 colony forming units (cfu) were counted. All experiments were conducted in triplicate and repeated at least three times on separate occasions.

### Honey pH and sugar content

The pH was measured in triplicate by dipping pH test strips (GE Healthcare, UK Ltd) into each honey and reading off the pH from the manufacturer’s colour chart. The percentage total sugar content of the honeys was estimated using a hand-held refractometer (TrustTechnology Co. Ltd., Shenzhen, China) in triplicate according to the manufacturer’s instructions. Sterile water was used as a blank.

### Extraction and measurement of total phenol content

Honey samples (2 × 10 g) were extracted with 20 mL of 80% (v/v) acetonitrile (ACN) containing 0.2% (v/v) formic acid (FA). Samples were vortexed for 30 s then extracted with end-over-end mixing on a blood tube rotator for 30 min at 4 °C. After centrifugation (10 min, 5^o^ C, 2500 g), supernatants were collected. The pellets were extracted once more, and the supernatants combined. The total phenol content of the extracts was measured by the Folin method [15].

### Solid Phase Extraction (SPE)

Honey extracts (~ 40 mL) were made up to 250 mL with 0.1% (v/v) FA in ultrapure water (UPW) and shaken manually to ensure dissolution. Briefly, each sample was applied to a solid phase extraction unit (Strata C18-E, GIGA SPE units, 10 g capacity; Phenomenex Ltd., UK) that had been prewashed in 0.1% (v/v) FA in ACN then equilibrated with 0.1% (v/v) FA in UPW. The unbound material, which contains organic acids and sugars, was collected. The columns were then washed with 2 volumes of UPW containing 0.1% FA. The bound extracts were eluted with 80% v/v ACN plus FA and aliquots were evaporated to dryness in a speed vacuum concentrator (Thermo Scientific, Waltham, USA). The total phenol content of bound and unbound fractions was measured as above. SPE was essential to prevent the high sugar content from obscuring the phytochemical diversity of the honeys.

### Examination of phytochemical profiles by non-targeted Liquid Chromatography – Mass Spectrometry (LC-MS)

The bound SPE fractions were concentrated 10-fold using a speed vacuum concentrator then re-suspended in 250 μL of 5% (v/v) ACN containing 0.1% (v/v) FA. These were filtered using Whatman syringeless filter devices (Mini-UniPrep™) then transferred into LC-MS vials. A Thermo Dionex U3000 pump, column oven and autosampler connected to a U3000 photodiode array detector (PDAD) (Thermo Fisher Scientific UK), was used for HPLC separations. The flow rate was 300 μL/min and 20 μL of extract was injected in part-loop mode. The column and guard column (Synergi C18 Hydro-RP 80 Ä, 150 × 2.0 mm (column) and 5.0 × 2.0 mm (guard), 4 μm particle size; Phenomenex Ltd.) were maintained at 30 °C. The solvents were A, HPLC grade water, and B, HPLC grade acetonitrile, acidified with 0.1% [v/v] formic acid. The gradient program was: 0–2 min hold 2% B, 2–5 min 2–5% B, 5–25 min 5–45% B; 25–26 min 45–100% B, 26–29 min hold at 100% B, 29–30 min 100–2% B, 30–35 min hold 2% B for re-equilibration. The eluent was monitored using the PDAD in absorbance mode over 200–600 nm and three UV channel set points at 280 nm, 365 nm and 520 nm). The HPLC eluent was then transferred to a Thermo LTQ-Orbitrap XL MS operated under Xcalibur software (Thermo Fisher Scientific UK) and from 2 to 30 mins the flow was directed to the ESI-MS. Data-dependent analysis applied a primary FT full scan from 100 to 2000 *m/z*, followed by a secondary LTQ-IT scan to collect MS^2^ fragmentation data on the top 3 most intense ions [16]. The samples were analyzed in negative mode with a randomized sample order with blank extracts analyzed at the start and end of each run. Specific honey samples were also analyzed in positive mode to confirm identities.

### Identification of components that correlate with antimicrobial activity

Peaks from MS chromatographs were selected using set detection criteria and processed using Xcalibur Quan software to obtain peak areas. Genstat software was used to correlate the abundance of the various components against antimicrobial activities of the honeys. Selected components that showed high correlation with antimicrobial activity were putatively identified using their MS data, exact mass and predicted formulae and MS^2^ properties.

### Statistical analysis

For the well diffusion and broth culture assay, data was compared using a two-tailed independent Student’s t-test. *P* values of ≤0.05 were considered statistically significant. Correlation coefficients (R^2^) for total phenol content, % total sugar content and pH with antimicrobial activity were calculated using Microsoft Excel, 2010. Genstat statistical software was used to assess the correlation of the abundance of the various components against the antimicrobial activities of the honeys.

## Results

### Background microbial contamination of honeys

No microbial growth was detected in any of the honeys 24 h after spreading the honey on Mueller Hinton Agar.

### Antimicrobial activity of Kazakhstan honeys using well diffusion assay

**Fig. 1:**
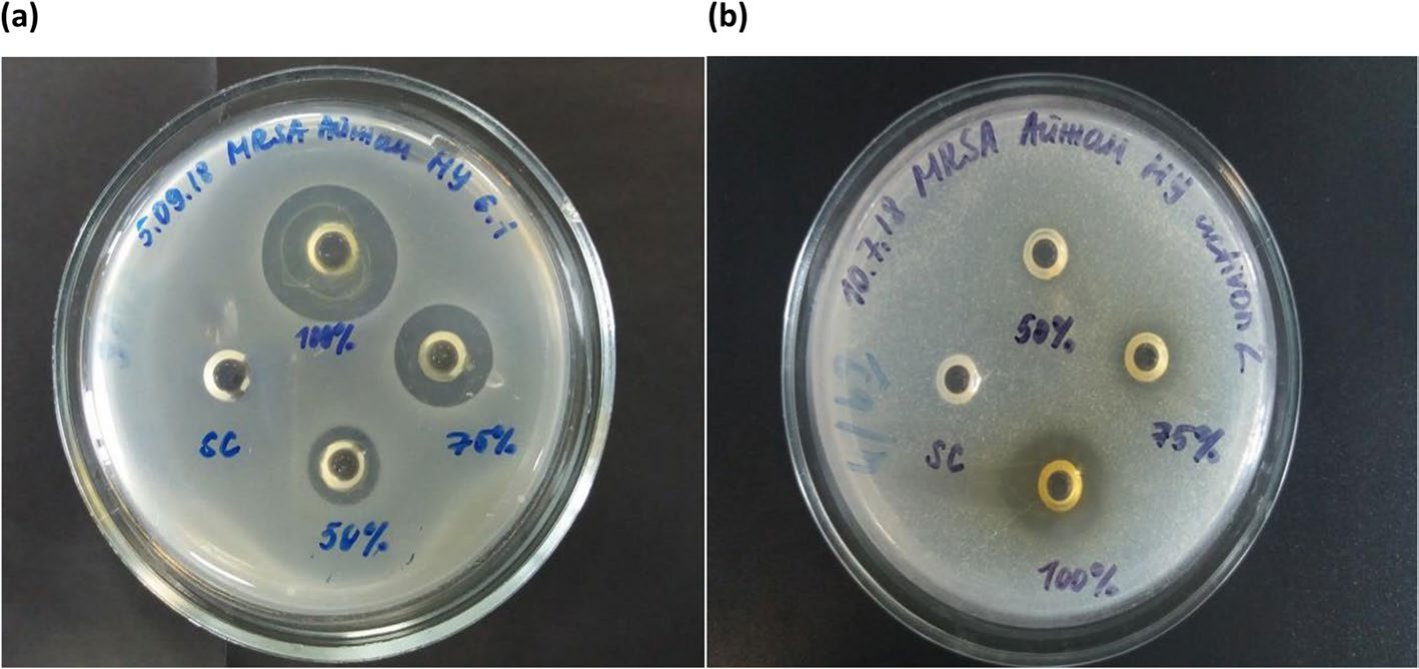
Antimicrobial activity of **(a)** buckwheat & multi-floral (honey 7) and **(b)** medical grade manuka (Activon) honey against a clinical isolate of MRSA. Samples tested at 100, 75 and 50% (w/v)

**Fig. 2:**
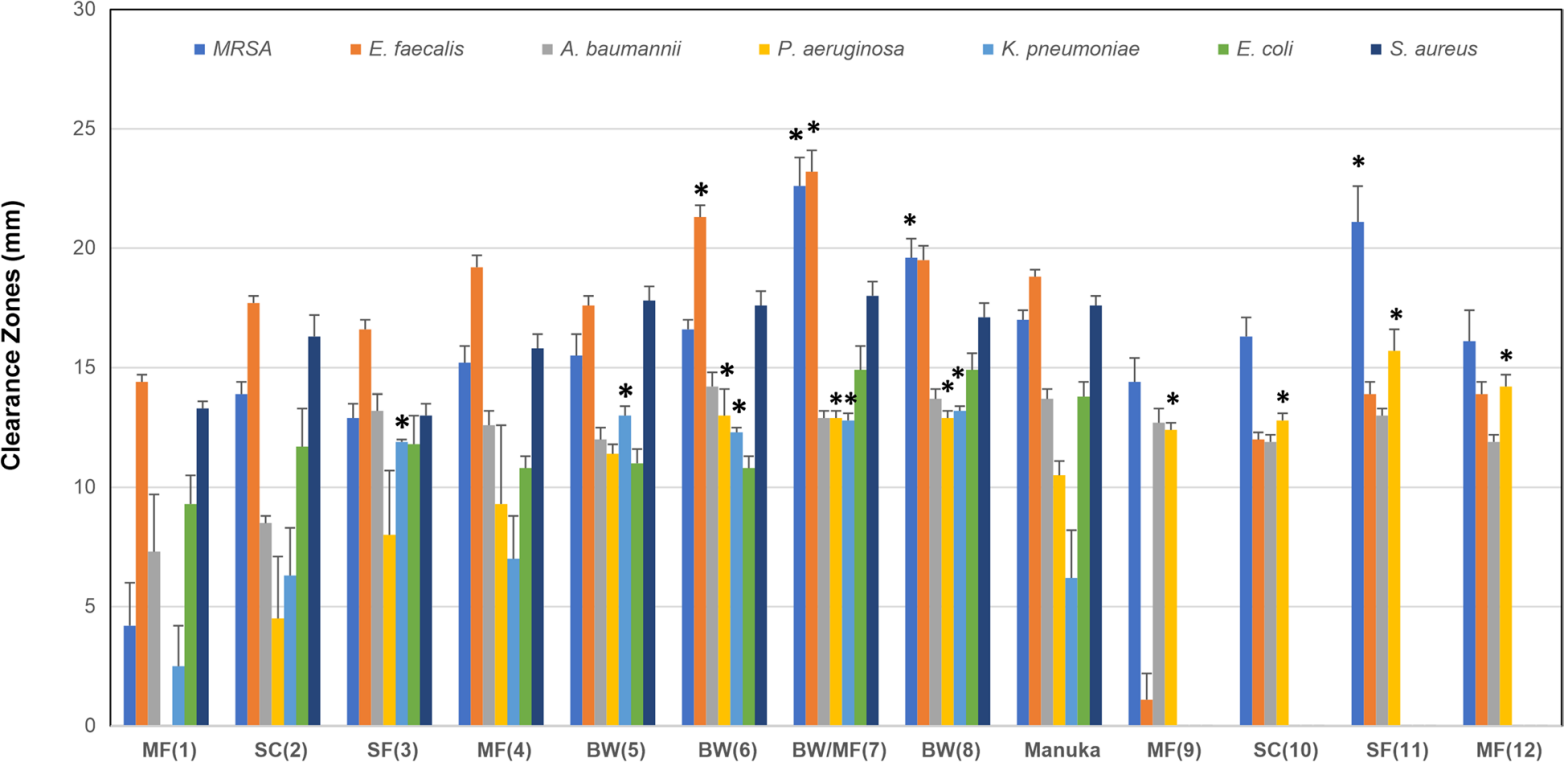
Antimicrobial activity of 12 Kazakhstan honeys and medical grade manuka honey at 100% concentration measured using agar well diffusion assay. Results are expressed as mean zone of inhibition (mm) ± standard error of the mean. This antimicrobial data was used for the correlation analysis. * - denotes higher antimicrobial activity than medical grade manuka honey (Activon) at *p* < 0.05. Each honey was given a unique number (1–12). Honeys 9–12 were not tested against all bacteria

The 100% concentrations of the honeys showed the largest zones of inhibition (Fig. 1). The micro-organisms that were most susceptible to the honeys were a clinical isolate of MRSA and *E. faecalis* (ATCC 19443). Honey 7 (buckwheat & multi-floral), honey 8 (buckwheat) and honey 11 (sunflower) demonstrated the highest antimicrobial activity against MRSA and were significantly higher than medical grade manuka honey (*p* < 0.05; Fig. 2). Honey 6 (buckwheat) and honey 7 (buckwheat & multi-floral) had higher antimicrobial activity against *E. faecalis* than medical grade manuka honey (*p* < 0.05). No anti-microbial activity against *C. albicans* and *M. furfur* was detected for any honey. The sugar control demonstrated a bacteriostatic effect against MRSA, *S. aureus* and *E. faecalis* without clear zones of inhibition. For *P. aeruginosa* and *A. baumannii,* there was an inhibitory effect of the sugar control, but this was lower than the more active honeys (6.2 mm and 2.6 mm respectively). The general susceptibility of *E. coli* to honeys has been noted before [17] and was illustrated by the anti-microbial effect of the sugar control (mean zone of inhibition = 10.0 ± 1.4 mm). Mean zone of inhibition values were sometimes less than 8 mm in the weaker honeys and sugar controls because zones of inhibition were not always present and were thus recorded as 0.

### Antimicrobial activity of honeys using broth culture assay

**Fig. 3:**
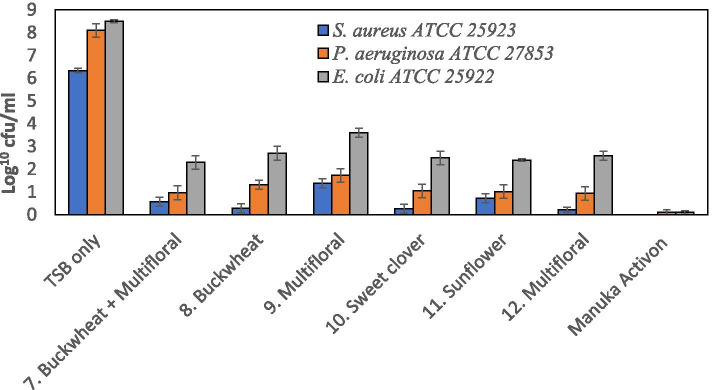
Anti-microbial activity of Kazakhstan honeys (7–12) and medical grade manuka honey (Activon) against *S. aureus*, *P. aeruginosa* and *E. coli* measured using a broth culture assay. All honeys produced significant reductions (*p* < 0.001)

Antibacterial activity of the 2nd batch of Kazakhstan honeys (7–12) and manuka honey against *S. aureus*, *P. aeruginosa* and *E. coli* was assessed using the broth culture assay (Fig. 3). Manuka honey had the highest anti-microbial activity with significant reduction in bacterial growth of all species (*p* < 0.05). All the Kazakhstan honeys demonstrated statistically significant anti-microbial activity against *S. aureus*, *P. aeruginosa* and *E. coli* when compared with the TSB control (*p* < 0.05). The sugar control reduced the growth of *S. aureus* and *E. coli* to 4–5 log^10^ cfu/ml compared to the TSB control (*p* < 0.05). However, the degree of inhibition caused by the sugar solutions was considerably less than the honeys (with minimum reduction to 3 log^10^ cfu/mL).

### Percentage sugar content, pH and Total Phenol Content (TPC) of the honeys

All the honeys contained a sugar content > 78%, with the highest at 84% (Table 1) and low pH (range from 2.7 to 3.8), consistent with previous reports [14]. Buckwheat honey (8) had the highest TPC followed by buckwheat honey (5), manuka honey, buckwheat & multifloral honey (7) and buckwheat honey (6), which were clearly higher than the other honeys. Interestingly, these were the same honeys that demonstrated the highest antimicrobial activity against MRSA and *E. faecalis.* They also had the darkest colour. However, sunflower honey (11) had good antimicrobial activity against MRSA but did not have a particularly high TPC.

**Table 1:** Percentage sugar content, pH and TPC of the Kazakhstan honeys and medical grade manuka honey

Honey	% sugar content ± SD	pH ± SD	TPC (mg/100mls) ± SD
**Multi-floral (1)**	83.7 ± 0.6	3.7 ± 0.3	129.3 ± 8.5
**Sweet clover (2)**	80.8 ± 0.3	3.7 ± 0.3	150.5 ± 17.7
**Sunflower (3)**	82.0 ± 0.0	2.7 ± 0.3	194.7 ± 0.4
**Multi-floral (4)**	78.3 ± 0.6	3.7 ± 0.3	193.9 ± 20.8
**Buckwheat (5)**	81.0 ± 1.0	3.2 ± 0.3	446.5 ± 9.1
**Buckwheat (6)**	–	3.2 ± 0.3	323.3 ± 7.4
**Buckwheat & multi-floral (7)**	81.3 ± 0.6	3.7 ± 0.3	329.1 ± 13.9
**Buckwheat (2nd batch 5) (8)**	80.3 ± 0.6	3.8 ± 0.3	718.6 ± 1.2
**Multi-floral (2nd batch 1) (9)**	82.0 ± 0.0	3.2 ± 0.3	213.3 ± 49.4
**Sweet clover (2nd batch 2) (10)**	81.0 ± 0.0	3.5 ± 0.0	149.3 ± 2.4
**Sunflower (2nd batch 3) (11)**	80.3 ± 0.3	3.5 ± 0.0	166.3 ± 0.9
**Multi-floral (2nd batch 4) (12)**	82.7 ± 0.6	3.5 ± 0.0	196.6 ± 2.4
**Manuka (Activon)**	81.0 ± 0.0	3.0 ± 0.0	450.3 ± 31.5

- Not tested

### Phytochemical profiling of Kazakhstan honeys by LC-MS

**Fig. 4:**
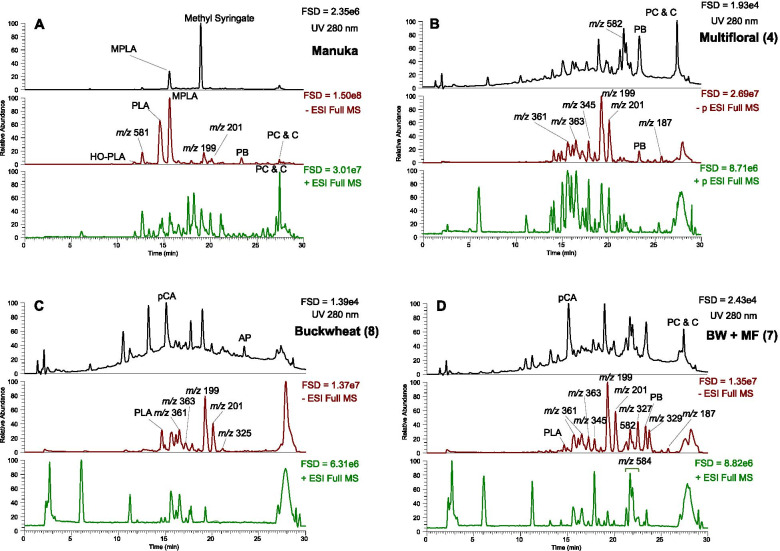
**A-D.** LC-MS profiles of selected Kazakhstan honey samples and medical grade manuka honey (Activon). The first panel represents UV trace at 280 nm, the second panel negative mode MS and the third panel positive mode MS profiles. BW + MF = buckwheat and multifloral honey. FSD = full scale deflection. PC = pinocembrin, C = chrysin, PB = pinobanksin, MPLA = methoxyphenyl lactic acid, PLA = phenyl lactic acid, HO-PLA = hydroxyphenyl lactic acid, pCA = *p*-coumaric acid

Each honey sample gave LC-MS profiles containing a range of components with *m/z* values and fragmentation (MS^2^) data that allowed putative identification against previous reports (Fig. 4A-D). Some abundant components were common to most, if not all, honey samples. For example, components with *m/z* [M-H]^−^ values of 363, 361, 201 and 199 were present in all samples but in different amounts. These are glucosides of decanedioic and decenedioic acids and the free acids respectively, as previously identified in honeys [15]. Some components were only present in a limited number of honey samples and certain components appeared to be characteristic of certain honeys. This is particularly apparent in the manuka samples which contained components such as methyl syringate, methoxyphenyl lactic acid and the maltosyl methyl syringate derivative (*m/z* [M-H]^−^ 581) unique to this monofloral honey [15, 18].

Although comparison of the LC-MS traces is informative, it was impossible to compare traces from multiple honey sources and correlate against their antimicrobial activities against the different microbes tested. Therefore, we took a non-targeted approach and quantified detectable peaks in the honey samples (Table S2) then correlated their abundance against antimicrobial activities. All honey samples were tested against MRSA, *E. faecalis*, *A. baumannii* and *P. aeruginosa* whereas a reduced set of honey samples were tested against *K. pneumonia*, *E. coli* and *S. aureus*. Interestingly, when these two sets of correlations were performed, components suggested to be antimicrobial were consistent across the two correlations (see supplementary material Table S3). Components that correlated with antimicrobial activity (Table 2) and certain components that were negatively correlated with antimicrobial activity, i.e. which appeared to promote bacterial growth, were identified (Table 3).

**Table 2:** MS properties and putative identification of honey components. Honey components correlated with antimicrobial activity

***m/z*** & RT	Exact mass	MS^**2**^	Formula as [M-H]^**−**^	Putative Identity
290.12 *m/z @* RT 12.70	290.1237	272, **230**, 200, 158	C_12_H_20_NO_7_	UK
151.04 *m/z @* RT 13.59	151.0398	107	C_8_H_7_O_3_	Hydroxyphenyl acetic acid
163.04 *m/z @* RT 15.06	163.0398	119	C_9_H_7_O_3_	*p*-coumaric acid
144.05 *m/z @* RT 17.73	144.0452	116, 86, **75**	C_9_H_6_NO	(1H)-quinolinone
309.13 *m/z @* RT 18.98	309.1336^a^	**263**	C_16_H_21_O_6_	Abscisic acid + FA adduct
263.13 *m/z @* RT 19.00	263.1282^a^	**219,** 204, 201, 186	C_15_H_19_O_4_	Abscisic acid
343.80 *m/z @* RT 19.75	343.2119	**325**, 307, 289, **229**, 209, 201, 171	C_18_H_31_O_6_	UK
325.18 *m/z @* RT 21.05	325.0709	281	C_18_H_29_O_5_	UK

Figures in bold are the major MS^2^ fragments, all molecular formula have error < 2 ppm

*FA* formic acid

^a^same peak

**Table 3:** MS properties and putative identification of honey components. Honey components negatively correlated with antimicrobial activity

***m/z*** & RT	Exact mass	MS^**2**^	Formula as [M-H]^**−**^	Putative Identity
285.13 *m/z @* RT 13.23	285.1338	**239**, 221, 217, **181**	C_13_H_20_O_4_	Unedone
225.11 *m/z @* RT 13.90	225.1129	207, **181**, 179, **165**, 163, 157, 147, 135, 113, 83	C_12_H_17_O_4_	UK
227.13 *m/z @* RT 14.68	227.1286	227, **183**, **165**, 163	C_12_H_19_O_4_	Difatty acid derivative
639.15 *m/z @* RT 14.87	639.1551	**477**, 459, **315**, 301	C_28_H_31_O_17_	Isorhamnetin-diglucoside
273.08 *m/z @* RT 18.86	273.0764	255, **167**, 163, 149	C_15_H_13_O_5_	Phloretin
395.19 *m/z @* RT 21.06	395.1916	**349**, 327, 187	C_17_H_31_O_10_	UK
582.26 *m/z @* RT 21.28 & RT 21.68	582.2596	**462**, 436, 342	C_34_H_36_N_3_O_6_	Tri-*p*-coumaroyl spermidine
301.20 *m/z @* RT 24.33	301.2014	**283**, 265, 221	C_16_H_29_O_5_	Hydroxy hexadecanedioic acid
287.22 *m/z @* RT 24.94	287.2224	**269**, 267, 251, 249, 241, 239, 223	C_16_H_31_O_4_	Dihydroxy palmitic acid

Figures in bold are the major MS^2^ fragments, all molecular formula have error < 2 ppm

## Discussion

In the well diffusion assay, the two Gram-positive micro-organisms, MRSA and *E. faecalis* were the most susceptible to the honeys tested. The Kazakhstan buckwheat & multifloral (7) honey displayed the highest antimicrobial activity against these two micro-organisms. It is clinically relevant that this honey is effective against MRSA because it is essential that new anti-microbials are found against antibiotic-resistant strains of this bacteria. Buckwheat & multifloral honey (7) and also buckwheat honey (8) from Kazakhstan could therefore be considered as a potential treatment for not only wound infections caused by *S. aureus* but also other disorders of the skin associated with *S. aureus* infection such as impetigo or impetiginized atopic dermatitis. The growth of *C. albicans* was not affected by any of the honeys tested in this study and this finding is consistent with reports in the scientific literature that *C. albicans* is more resistant to honey than many other microbial species e.g. [19]. The honeys tested in this study also did not exhibit anti-fungal activity against *M. furfur*, therefore it is unlikely that they would be efficacious for the treatment of skin diseases caused by *C. albicans* or *M. furfur*.

All honeys tested were significantly bactericidal against *S. aureus*, *P. aeruginosa* and *E. coli* as assessed by the broth culture assay. Indeed, using this assay, manuka honey was more effective than the buckwheat & multifloral honey (7), which was more effective than manuka in the well diffusion assay. This disparity may be related to the ease by which the different chemical components responsible for the anti-bacterial activities of the honeys can diffuse through the nutrient agar in the well diffusion assay. It has previously been suggested that the anti-microbial components of honey can move better in liquid broth than in agar [6, 14]. However, the use of different bacterial strains in the different assays may also be a factor.

Comparing the chemical composition of the honeys against their anti-microbial activity may indicate components responsible for the anti-microbial activity. Higher total sugar content was not associated with increased antimicrobial activity as correlation coefficients (R^2^) for % total sugar content and antibacterial activity against MRSA and *E. faecalis,* for example, were − 0.54 and − 0.35 respectively. The pH of the honeys ranged from 2.7 to 3.8 and did not correlate with antibacterial activity against MRSA and *E. faecalis* (R^2^ = 0.10 and 0.17 respectively; well diffusion data). The most effective honeys i.e. manuka honey, buckwheat honey (8) and buckwheat & multifloral honey (7) had the highest TPC and R^2^ values for TPC and activity against MRSA and *E. faecalis* were 0.42 (*p* = 0.14) and 0.40 (*p* = 0.18), respectively. This agrees with Kaskoniene et al., [20] and Fyfe et al. [15] who showed that the more effective honeys had higher polyphenol levels. However, like our findings, Fyfe et al., [15] did not find a statistically significant correlation between TPC and antimicrobial activity. Although TPC may be significant, the actual polyphenol composition of honeys may be more important, and this may explain why sunflower honey (11) was not particularly high in TPC but demonstrated good antimicrobial activity against MRSA. We did not measure the hydrogen peroxide content of the honeys, however Fyfe et al. [15] reported that hydrogen peroxide content of Scottish honeys did not differ between the effective and non-effective honeys.

Interestingly, correlation analysis also revealed that honeys that were effective against MRSA were also effective against *P. aeruginosa* (R^2^ = 0.85) and honeys that were effective against *E. faecalis* were also effective against *K. pneumoniae* (R^2^ = 0.86). There was a lower but still significant correlation (R^2^ = 0.55) between effectiveness against *P. aeruginosa* and *A. baumannii*. These correlations were not related to the bacteria being Gram-positive or Gram-negative but are likely related to the specific mechanism of action of the antibacterial components present in the honeys and the bacterial molecules they act upon. Further research is required to determine what these mechanisms are.

The correlation of composition with antimicrobial activity indicated candidate components including hydroxyphenyl acetic acid, *p*-coumaric acid, (1H)-quinolinone, and abscisic acid (Table 2). Hydroxyphenyl acetic acid has previously been identified in other types of honey and has been reported to have antibacterial activity against *P. aeruginosa* [21, 22]. Huberman et al., [22] suggested that the antibacterial activity of phenols was related to the production of free radicals which can exert an inhibitory effect on microbial proteins. Antibacterial activity of *p*-coumaric acid has been reported against *S. aureus* and *E. coli* [23] and was suggested to act by increasing bacterial membrane permeability, binding to DNA, and altering gene expression and bacterial replication. *p*-Coumaric acid has been reported to be more abundant in buckwheat honey than in manuka honey [24]. 1H-quinolinones have previously been found in honey [25] and have been shown to have antibacterial activity with their mechanism of action related to inhibition of bacterial DNA gyrase [26]. Abscisic acid (ABA) is a plant hormone which has been found in honey [15] and ABA isolated from Korean acacia honey was reported to have antibacterial activity against *Helicobacter pylori* [27].

On the other hand, compounds identified as potentially supporting the growth of bacteria were also identified. Unedone has previously been identified in honeys [28] and may be derived from ABA. Isorhamnetin-diglucoside is a flavonol derivative and has also been detected in other types of honey [9, 28]. There are no previous reports on the antibacterial effectiveness of these two compounds. Phloretin, a dihydrochalcone, has been shown to have antibacterial activity against *Propionibacterium acnes*, *S. aureus*, *E. coli* and *P. aeruginosa*. The antibacterial activity of phloretin was reportedly strain specific and in *P. acnes* may arise through binding to β-ketoacyl acyl carrier protein (ACP) synthase III (KAS III) and interference with fatty acid synthesis [29], or in *S. aureus*, it may interfere with fatty acid and sugar utilization [30]. Phloretin from natural sources has been shown to be bound to other compounds [31] and this may alter its antibacterial activity. The presence of Tri-*p*-coumaroyl spermidine has been noted in honeys [32] but its antimicrobial potential has not been reported. Fatty acids in honey such as hydroxy hexadecanedioic acid, dihydroxy palmitic acid [17] and the undefined difatty acid derivative may provide fuel for bacterial growth or provide protection from other antibacterial agents in the honeys [33].

A limitation of this study is that we did not compare the antimicrobial activity of the honeys with appropriate antibiotics serving as positive controls. This could have provided important information about the effectiveness of the honeys in comparison to antibiotics.

In the future, it would be important to investigate the anti-microbial properties of the honeys against micro-organisms associated with other skin disorders such as *P. acnes*, determine the minimal inhibitory concentration and hydrogen peroxide content of the honeys, and investigate the antimicrobial properties and mechanisms of the components identified as correlating with antimicrobial activity.

## Conclusions

The Kazakhstan honeys tested in this study have antimicrobial activity against wound and skin infecting microorganisms. The antimicrobial compounds identified in this study could be considered as bioactive agents for potential therapeutic use in the treatment of wound or skin infections and should be investigated further.

## Supplementary Information

**Additional file 1.**

**Additional file 2: Table S1.** a. *E. coli* ATCC 25922 antibiotic susceptibility testing (antibiogram). b. *P. aeruginosa* ATCC 27853 antibiotic susceptibility testing (antibiogram). c. *S. aureus* ATCC 25923 antibiotic susceptibility testing (antibiogram). **Table S2.** Peak areas for all detected peaks in honey samples. **Table S3.** Correlation of peak abundance with antimicrobial activity against different bacteria.

## Data Availability

Some of the data generated or analysed during the study are included in this publication in supplementary information files. Other data analysed are available on reasonable request from the corresponding author.

## Abbreviations

ABA: Abscisic acid
ACN: Acetonitrile
ACP: Acyl carrier protein
ATCC: American type culture collection
BW + MF: Buckwheat & multifloral
C: Chrysin
*C. albicans*: *Candida albicans*
*E. coli*: *Escherichia coli*
ESI-MS: Electrospray ionisation mass spectrometry
FA: Formic acid
FSD: Full scale deflection
FT: Fourier transform
HO-PLA: Hydroxyphenyl lactic acid
HPLC: High performance liquid chromatography
KAS III: β-ketoacyl acyl carrier protein (ACP) synthase III
LC-MS: Liquid chromatographic mass spectrometry
*M. furfur*: *Malassezia furfur*
MGO: Methylglyoxal
MPLA: Methoxyphenyl lactic acid
MRSA: Methicillin resistant *Staphylococcus aureus*
*P. aeruginosa*: *Pseudomonas aeruginosa*
PB: Pinobanksin
PBS: Phosphate buffered saline
PC: Pinocembrin
PDAD: Photodiode array detector
PLA: Phenyl lactic acid
*S. aureus*: *Staphylococcus aureus*
SPE: Solid phase extraction
TPC: Total phenol content
TSB: Tryptone soya broth
UK: Unknown
UPW: Ultrapure water
UV: Ultraviolet
