# Supplementary material for: Identification of components in Kazakhstan honeys that correlate with antimicrobial activity against wound and skin infecting microorganisms
Source: BMC Complement Med Ther. 2021 Dec 20;21:300. doi: 10.1186/s12906-021-03466-0 (PMC8690519; doi:10.1186/s12906-021-03466-0)
Supplement: Supplementary file 2 — Additional file 2: Table S1. a. E. coli ATCC 25922 antibiotic susceptibility testing (antibiogram). b. P. aeruginosa ATCC 27853 antibiotic susceptibility testing (antibiogram). c. S. aureus ATCC 25923 antibiotic susceptibility testing (antibiogram). Table S2. Peak areas for all detected peaks in honey samples. Table S3. Correlation of peak abundance with antimicrobial activity against different bacteria. [file 12906_2021_3466_MOESM2_ESM.docx]

**SUPPLEMENTARY DATA**

**Table S1a. *E. coli* ATCC 25922 antibiotic susceptibility testing (antibiogram)**

| **Antibiotic** | **Symbol** | **Disc dosage** | **# of lot** | **Zone of inhibition diameter range (mm)** | **Zone of Inhibition Result 1 (mm)** | **Zone of Inhibition Result 2 (mm)** | **Zone of Inhibition Result 3 (mm)** | **Zone of Inhibition Result 4 (mm)** | **Susceptibility** |
| --- | --- | --- | --- | --- | --- | --- | --- | --- | --- |
| Cotrimoxazole | COT | 25 µg | 277518 | 23-29 | 25 | 26 | 27 | 26 | Sensitive |
| Aztreonam | AT | 30 µg | 277219 | 28-36 | 32 | 31 | 34 | 32 | Sensitive |
| Moxifloxacin | MO | 5 µg | 278532 | 28-35 | 31 | 32 | 30 | 31 | Sensitive |
| Ampicillin | AMP | 10 µg | 303080 | 15-22 | 17 | 18 | 20 | 19 | Sensitive |
| Amikacin | AK | 30 µg | 277016 | 19-26 | 23 | 22 | 21 | 23 | Sensitive |
| Ofloxacin | OF | 5 µg | 276582 | 29-33 | 31 | 32 | 30 | 31 | Sensitive |
| Ampicillin/Sulbactam | A/S | 10/10 µg | 277304 | 19-24 | 21 | 22 | 21 | 21 | Sensitive |
| Norfloxacin | NX | 10 µg | 303176 | 28-35 | 31 | 32 | 33 | 31 | Sensitive |
| Nitrofurantoin | NIT | 300 µg | 302484 | 20-25 | 23 | 22 | 21 | 23 | Sensitive |
| Tetracycline | TE | 30 µg | 278332 | 18-25 | 21 | 22 | 20 | 21 | Sensitive |
| Trimethoprim | TR | 5 µg | 246861 | 21-28 | 26 | 25 | 23 | 24 | Sensitive |
| Doxycycline | DO | 30 µg | 302484 | 18-24 | 20 | 21 | 22 | 20 | Sensitive |

**Table S1b. *P. aeruginosa* ATCC 27853 antibiotic susceptibility testing (antibiogram)**

| **Antibiotic** | **Symbol** | **Disc dosage** | **# of lot** | **Zone of inhibition diameter range (mm)** | **Zone of inhibition Result 1 (mm)** | **Zone of inhibition Result 2 (mm)** | **Zone of inhibition Result 3 (mm)** | **Zone of inhibition Result 4 (mm)** | **Susceptibility** |
| --- | --- | --- | --- | --- | --- | --- | --- | --- | --- |
| Imipenem | IPM | 10 µg | 303314 | 20-28 | 21 | 25 | 24 | 24 | Sensitive |
| Ticarcillin/Clavulanate | TCC | 75/10 µg | 277104 | 20-28 | 25 | 26 | 23 | 23 | Sensitive |
| Piperacillin/Tazobactam | PIT | 100/10 µg | 277752 | 25-33 | 30 | 30 | 31 | 31 | Sensitive |
| Gentamicin | GEN | 50 µg | 294771 | 20-25 | 24 | 24 | 23 | 24 | Sensitive |
| Ceftriaxone | CTR | 30 µg | 277614 | 17-23 | 20 | 20 | 21 | 22 | Sensitive |
| Piperacillin | PI | 100 µg | 277102 | 25-33 | 30 | 31 | 31 | 30 | Sensitive |
| Ciprofloxacin | CIP | 5 µg | 303177 | 25-33 | 31 | 31 | 30 | 31 | Sensitive |
| Lomefloxacin | LOM | 10 µg | 244836 | 22-28 | 26 | 27 | 24 | 26 | Sensitive |
| Meropenem | MRP | 10 µg | 277310 | 27-33 | 30 | 24 | 25 | 29 | Sensitive |
| Ceftazidime | CAZ | 30 µg | 217101 | 22-29 | 23 | 20 | 30 | 28 | Sensitive |
| Ticarcillin | TI | 75 µg | 277413 | 21-27 | 25 | 25 | 24 | 25 | Sensitive |

**Table S1c. *S. aureus* ATCC 25923 antibiotic susceptibility testing (antibiogram)**

| **Antibiotic** | **Symbol** | **Disc dosage** | **# of lot** | **Zone of inhibition diameter range (mm)** | **Zone of inhibition Result 1 (mm)** | **Zone of inhibition Result 2 (mm)** | **Zone of inhibition Result 3 (mm)** | **Zone of inhibition Result 4 (mm)** | **Susceptibility** |
| --- | --- | --- | --- | --- | --- | --- | --- | --- | --- |
| Oxacillin | OX | 1 µg | 276411 | 18-24 | 22 | 21 | 22 | 22 | Sensitive |
| Amoxacillin/Clavulanate | AMC | 30 µg | 277926 | 28-36 | 30 | 30 | 31 | 31 | Sensitive |
| Clindamycin | CD | 2 µg | 276922 | 24-30 | 25 | 25 | 25 | 26 | Sensitive |
| Azithromycin | AZM | 15 µg | 247087 | 21-26 | 23 | 22 | 22 | 23 | Sensitive |
| Linezolid | LZ | 30 µg | 278247 | 25-32 | 27 | 28 | 28 | 28 | Sensitive |
| Penicillin | P | 10 µg | 284644 | 26-37 | 28 | 28 | 28 | 28 | Sensitive |
| Erythromycin | E | 15 µg | 233971 | 22-30 | 25 | 24 | 25 | 25 | Sensitive |
| Clarithromycin | CLR | 15 µg | 247205 | 26-32 | 27 | 27 | 27 | 28 | Sensitive |
| Ertapenem | ETP | 10 µg | 276931 | 24-31 | 26 | 26 | 26 | 26 | Sensitive |
| Cefoxatime | CTX | 30 µg | 276266 | 25-31 | 27 | 27 | 27 | 27 | Sensitive |
| Chloramphenicol | C | 30 µg | 276406 | 19-26 | 22 | 22 | 22 | 22 | Sensitive |
| Cefepime | CPM | 30 µg | 278070 | 23-29 | 25 | 25 | 25 | 25 | Sensitive |
| Vancomycin | VA | 30 µg | 276578 | 17-21 | 19 | 19 | 19 | 19 | Sensitive |
| Cefazolin | CZ | 30 µg | 278166 | 29-35 | 30 | 30 | 30 | 30 | Sensitive |
| Levofloxacin | LE | 5 µg | 278248 | 25-30 | 27 | 28 | 28 | 28 | Sensitive |
| Cefoxitin | CX | 30 µg | 277742 | 23-29 | 26 | 27 | 27 | 27 | Sensitive |

**Table S2. Peak areas for all detected peaks in honey samples**

| ***m/z* @ RT (min)** | **Multi-floral (1)** | **Sweet Clover (2)** | **Sunflower (3)** | **Multi-floral (4)** | **Buckwheat (5)** | **Buckwheat (6)** | **Buckwheat & Multifloral (7)** | **Buckwheat (2nd batch 8)** | **Multi-floral (9)** | **Sweet Clover (10)** | **Sunflower (11)** | **Multi-floral (12)** | **Manuka** |
| --- | --- | --- | --- | --- | --- | --- | --- | --- | --- | --- | --- | --- | --- |
| **581.17 *m/z* @12.64** | - | - | - | - | - | - | - | - | 1,321,844 | 1,681,450 | 5,314,029 | - | 393,460,362 |
| **290.12 *m/z* @12.70** | - | 15,382,535 | - | - | 492,948 | 25,699,413 | - | - | - | 59,044,328 | 98,855,569 | 3,510,023 | - |
| **165.06 *m/z* @12.49** | 3,115,756 | 2,500,174 | 9,460,104 | 2,731,672 | 8,456,728 | 803,637 | 4,846,613 | 1,643,900 | 1,352,480 | 3,958,430 | 4,106,600 | 5,432,019 | 5,455,990 |
| **177.02 *m/z* @12.73** | 3,093,905 | 2,396,174 | 9,242,430 | 2,726,767 | 8,497,208 | 769,854 | 4,860,613 | 1,617,895 | 1,345,462 | 3,953,073 | 4,122,860 | 5,462,695 | 5,109,669 |
| **153.02 *m/z* @12.84** | 2,970,912 | 2,323,944 | 8,564,129 | 2,445,209 | 3,001,192 | 262,190 | 1,563,925 | 3,192,802 | 1,105,647 | 1,872,727 | 4,038,158 | 3,028,381 | 650,156 |
| **236.09 *m/z* @13.15** | - | 2,046,104 | 18,837,429 | - | 2,437,710 | 337,635 | 3,908,121 | - | - | 1,643,610 | 2,133,032 | 6,348,934 | - |
| **285.13 *m/z* @13.23** | 6,151,602 | - | 1,725,492 | 5,139,558 | 1,726,986 | - | - | 3,283,654 | 1,004,193 | - | - | - | - |
| **365.18 *m/z* @13.43** | 2,326,846 | 12,481,291 | 16,149,664 | 1,924,607 | 5,488,554 | 7,073,274 | 4,833,670 | 2,111,448 | 13,396,996 | 6,103,891 | 15,388,734 | 1,731,219 | - |
| **151.04 *m/z* @13.59** | - | 6,366,626 | 649,200 | - | 2,052,313 | 5,366,627 | 437,495 | 158,148 | 326,521 | 11,658,579 | 12,311,399 | 2,372,350 | 2,836,833 |
| **569.21 *m/z* @13.62** | 8,842,964 | 29,770,629 | 54,826,040 | 7,548,353 | 17,868,157 | 12,473,316 | 18,891,071 | 7,356,531 | 21,284,689 | 10,147,659 | 26,223,196 | 6,748,873 | 10,813,650 |
| **225.11 *m/z* @13.90** | 20,372,580 | 9,093,284 | 22,867,991 | 17,501,036 | 8,447,156 | 1,584,909 | 3,876,232 | 15,419,107 | 8,984,595 | 1,951,696 | 2,704,785 | 6,055,151 | 17,559,647 |
| **317.03 *m/z* @14.01** | - | 2,017,725 | 15,886,374 | - | 4,908,169 | - | 5,520,409 | - | 1,325,263 | - | - | 6,251,280 | - |
| **419.12 *m/z* @14.13** | 1,779,489 | 7,831,052 | - | 1,071,947 | - | 819,670 | 813,137 | 7,603,157 | 55,959,236 | 1,482,126 | 2,668,408 | 3,969,053 | 9,775,931 |
| **173.08 *m/z* @14.38** | 8,344,289 | 13,943,418 | 23,496,814 | 6,384,299 | 14,792,776 | 6,629,257 | 10,077,693 | 4,598,661 | 13,289,238 | 14,975,450 | 21,567,876 | 6,760,893 | 4,549,791 |
| **377.18 *m/z* @14.46** | 11,669,703 | 29,597,196 | 27,663,810 | 16,097,282 | 14,055,666 | 15,541,010 | 7,448,902 | 32,520,888 | 10,038,793 | 15,406,994 | 30,207,532 | 15,623,011 | - |
| **165.05 *m/z* @14.66** | 3,667,638 | 186,350,007 | 1,239,901,040 | 6,199,809 | 64,925,576 | 69,335,870 | 21,061,689 | 8,743,995 | 54,561,884 | 28,685,464 | 27,794,933 | 23,624,199 | 2,335,759,600 |
| **227.13 *m/z* @14.68** | 5,330,826 | 4,475,477 | - | 5,108,350 | 942,872 | 413,365 | - | 3,695,117 | 4,011,200 | - | - | - | - |
| **639.15 *m/z* @14.87** | 3,913,827 | 2,103,947 | 5,174,507 | 3,728,031 | 1,304,247 | 920,696 | - | 4,272,324 | 2,993,000 | 474,992 | 1,424,641 | 904,831 | - |
| **203.13 *m/z* @14.98** | 6,933,125 | 44,641,270 | 58,517,028 | 3,658,061 | 24,204,360 | 15,869,467 | 29,495,598 | 6,074,546 | 50,784,881 | 38,564,906 | 56,554,725 | 11,462,198 | 9,068,417 |
| **163.04 *m/z* @15.06** | 2,317,093 | 20,047,357 | 4,757,790 | 1,841,071 | 2,947,306 | 5,854,599 | 1,778,954 | 1,794,219 | 7,402,873 | 55,049,644 | 63,358,641 | 10,937,916 | 5,197,847 |
| **361.15 *m/z* @15.17** | 10,274,563 | 27,009,228 | 51,148,298 | 7,597,936 | 18,753,906 | 8,913,587 | 17,687,157 | 7,241,809 | 22,351,270 | 13,481,728 | 23,405,401 | 7,321,716 | 16,986,549 |
| **369.19 *m/z* @15.28** | 2,677,876 | 3,207,286 | 5,915,990 | 3,167,590 | 5,458,146 | 603,591 | 1,575,696 | 10,885,619 | 899,013 | 670,376 | 1,261,453 | 282,339 | - |
| **391.14 *m/z* @15.58** | - | - | - | - | - | - | - | - | - | - | - | - | 2,495,157,018 |
| **919.23 *m/z* @15.63** | 80,873 | - | - | - | - | - | - | 403,360 | 73,809,343 | - | - | - | - |
| **523.20 *m/z* @15.66** | 35,659,612 | 49,564,019 | 100,578,113 | 29,021,830 | 34,432,813 | 23,961,527 | 24,198,916 | 27,845,332 | 37,247,816 | 26,985,190 | 49,137,897 | 10,159,755 | 47,880,981 |
| **361.15 *m/z* @15.69** | 99,523,281 | 218,778,642 | 333,673,512 | 80,702,986 | 167,074,061 | 90,489,255 | 156,209,511 | 73,564,896 | 174,343,800 | 125,491,245 | 205,759,452 | 76,039,829 | 137,482,599 |
| **361.15 *m/z* @16.13** | 63,267,575 | 112,875,185 | 241,225,922 | 45,833,080 | 96,664,326 | 52,161,422 | 97,937,793 | 35,426,947 | 92,038,322 | 68,293,720 | 111,083,579 | 40,128,856 | 89,749,140 |
| **363.17 *m/z* @16.38** | 39,047,539 | 82,414,589 | 120,919,759 | 34,780,120 | 63,802,625 | 32,523,472 | 61,654,666 | 29,552,387 | 74,239,890 | 49,810,595 | 74,940,988 | 35,192,269 | 54,675,820 |
| **361.15 *m/z* @16.52** | 99,686,438 | 214,330,169 | 354,790,871 | 82,161,435 | 156,002,066 | 86,530,374 | 157,092,350 | 72,193,070 | 187,547,142 | 127,478,811 | 200,025,196 | 73,989,490 | 155,770,530 |
| **363.17 *m/z* @16.77** | 24,310,736 | 51,277,810 | 70,102,837 | 18,103,519 | 40,805,289 | 18,031,410 | 40,210,113 | 16,467,823 | 41,519,642 | 28,425,108 | 42,490,921 | 19,316,328 | 34,072,494 |
| **347.17 *m/z* @16.84** | 53,548,891 | 44,845,023 | 88,024,564 | 35,436,633 | 28,532,418 | 16,963,131 | 40,648,606 | 42,231,859 | 76,685,147 | 56,346,628 | 70,153,416 | 22,001,098 | 21,985,063 |
| **165.06 *m/z* @17.07** | - | - | - | - | - | - | - | - | - | - | - | - | 19,703,643 |
| **363.17 *m/z* @17.16** | 53,938,424 | 136,368,505 | 238,545,298 | 48,951,521 | 97,071,801 | 41,687,902 | 96,088,978 | 38,246,967 | 113,618,398 | 71,635,974 | 112,013,448 | 43,301,692 | 85,304,996 |
| **187.10 *m/z* @17.23** | 23,107,257 | 22,842,452 | 40,053,674 | 15,517,628 | 23,942,389 | 9,058,394 | 15,408,901 | 13,572,295 | 25,934,256 | 19,408,950 | 23,503,162 | 41,951,135 | 26,136,043 |
| **349.19 *m/z* @17.44** | 23,110,556 | 25,503,076 | 53,302,887 | 13,105,113 | 13,097,011 | 7,194,914 | 19,604,956 | 19,457,166 | 53,241,356 | 30,126,983 | 34,019,078 | 9,435,980 | 10,527,149 |
| **187.10 *m/z* @17.66** | 5,393,143 | 4,818,796 | 8,992,450 | 3,899,732 | 5,417,440 | 2,324,468 | 5,681,516 | 3,276,976 | 6,063,821 | 3,736,851 | 4,701,393 | 8,925,122 | 5,578,072 |
| **144.05 *m/z* @17.73** | 235,614 | 28,070,707 | 2,283,102 | 153,040 | 2,518,376 | 10,838,250 | 1,013,143 | 639,348 | 2,426,765 | 18,616,055 | 18,522,505 | 8,218,432 | 644,578 |
| **345.15 *m/z* @17.88** | 79,765,611 | 92,034,830 | 119,760,287 | 53,860,475 | 137,501,570 | 28,615,131 | 107,031,384 | 49,030,294 | 145,070,872 | 84,923,572 | 81,977,421 | 41,437,326 | 94,322,305 |
| **479.25 *m/z* @17.93** | - | - | - | - | - | - | - | - | - | - | - | - | 104,892,913 |
| **273.08 *m/z* @18.17** | 1,032,571 | 2,293,347 | 2,881,401 | 961,851 | 1,472,860 | 662,824 | 1,688,777 | 1,124,963 | 1,272,311 | - | - | 1,672,109 | 1,778,336 |
| **347.17 *m/z* @18.53** | 45,655,617 | 34,616,255 | 42,686,089 | 29,912,646 | 53,202,679 | 11,067,489 | 32,291,256 | 24,621,268 | 58,811,992 | 33,264,210 | 29,911,260 | 14,533,463 | 29,423,774 |
| **849.36 *m/z* @18.68** | 5,024,670 | 411,686 | 10,068,915 | 4,751,151 | 1,245,953 | - | 150,119 | 10,498,595 | 2,077,427 | 695,335 | 634,158 | 80,667 | - |
| **273.08 *m/z* @18.86** | 5,748,877 | - | - | 3,880,870 | 789,251 | - | - | 2,518,768 | 701,682 | - | - | - | - |
| **309.13 *m/z* @18.98** | 3,666,716 | 14,260,579 | 2,588,303 | 2,518,373 | 2,899,955 | 3,441,836 | 3,294,520 | 4,102,812 | 11,303,127 | 22,865,726 | 31,858,113 | 4,970,960 | 69,153,523 |
| **263.13 *m/z* @19.00** | 1,828,909 | 6,956,362 | 1,280,889 | 1,199,840 | 1,235,411 | 1,914,336 | 1,590,269 | 2,203,109 | 5,948,901 | 10,799,517 | 15,703,738 | 2,712,740 | 35,392,311 |
| **199.10 *m/z* @19.24** | 358,493,929 | 535,240,332 | 685,648,877 | 224,810,601 | 470,907,758 | 200,048,051 | 413,114,950 | 232,722,930 | 591,532,386 | 631,435,761 | 509,223,957 | 245,097,950 | 464,023,314 |
| **343.80 *m/z* @19.75** | - | 1,055,795 | 3,569,762 | - | 1,354,095 | 432,432 | 901,333 | - | 905,755 | 2,028,785 | 2,514,636 | 1,897,101 | 112,768,875 |
| **263.13 *m/z* @19.94** | 13,254,468 | 35,588,386 | 11,004,306 | 7,572,247 | 8,401,882 | 5,294,345 | 10,406,918 | 15,138,745 | 23,471,377 | 37,248,603 | 36,908,703 | 11,195,928 | 69,703,643 |
| **201.11 *m/z* @20.11** | 158,065,139 | 252,398,475 | 308,392,073 | 104,611,790 | 219,615,583 | 89,444,402 | 204,252,801 | 103,867,904 | 282,149,019 | 278,696,806 | 240,878,558 | 120,368,388 | 186,936,144 |
| **349.19 *m/z* @20.57** | 8,071,557 | 6,085,142 | 7,095,899 | 4,057,539 | 4,364,819 | 1,515,471 | 4,434,548 | 3,944,909 | 11,167,185 | 5,400,643 | 4,547,813 | 1,876,097 | 3,775,748 |
| **687.31 *m/z* @20.59** | 5,728,401 | 1,928,915 | 6,393,691 | 5,719,858 | 29,734,241 | 73,537 | 1,967,577 | 15,492,369 | 2,833,568 | 2,601,191 | 2,057,764 | - | - |
| **463.25 *m/z* @20.76** | 6,111,826 | - | - | 6,723,229 | - | - | - | 6,438,967 | - | - | 3,549,683 | - | - |
| **289.04 *m/z* @20.80** | - | - | - | - | - | - | - | - | - | - | - | - | 47,659,505 |
| **325.18 *m/z* @21.05** | - | 39,836,534 | - | - | 7,232,067 | 13,338,638 | 4,338,937 | - | - | 156,126,715 | 127,952,686 | 26,485,435 | - |
| **395.19 *m/z* @21.06** | 9,961,871 | 13,718,642 | 20,891,599 | 5,054,756 | 12,017,384 | 2,864,673 | 18,200,014 | 6,480,629 | 21,024,270 | 9,199,292 | 7,242,504 | 4,908,294 | 6,794,130 |
| **489.19 *m/z* @21.10** | - | - | - | - | - | - | - | - | - | - | - | - | 50,509,076 |
| **582.26 *m/z* @21.28** | 87,181,208 | 6,646,961 | 117,316,375 | 46,216,741 | 45,356,835 | 1,108,074 | 45,603,246 | 51,746,059 | 25,734,000 | 3,206,430 | 2,577,278 | 27,172,063 | 2,195,641 |
| **285.80 *m/z* @21.33** | 6,132,245 | 2,864,753 | 14,411,860 | 2,905,438 | 5,066,198 | - | 3,383,355 | 2,864,229 | 5,706,648 | 6,071,551 | 4,700,071 | 5,770,148 | 89,674,407 |
| **582.26 *m/z* @21.68** | 102,722,428 | 33,546,710 | 112,136,151 | 53,413,090 | 69,414,342 | 3,139,204 | 63,044,515 | 75,340,048 | 32,296,675 | 24,663,079 | 20,065,732 | 85,759,761 | 4,259,180 |
| **628.27 *m/z* @21.94** | 25,990,784 | 17,726,357 | 25,543,368 | 12,063,953 | 28,727,729 | 1,138,077 | 13,140,276 | 20,305,442 | 9,981,661 | 16,646,056 | 12,835,191 | 33,309,641 | 1,558,207 |
| **628.27 *m/z* @22.43** | 7,119,254 | 5,555,475 | 12,079,043 | 3,728,807 | 9,737,934 | 287,802 | 1,973,228 | 8,089,152 | 5,865,014 | 8,464,932 | 6,341,738 | 6,562,419 | 3,127,593 |
| **327.22 *m/z* @22.51** | 5,511,162 | 13,269,597 | 110,500,365 | 3,964,365 | 37,874,400 | 4,116,689 | 36,280,917 | 8,532,163 | 7,142,343 | 12,812,281 | 13,418,914 | 95,768,149 | 10,723,298 |
| **271.06 *m/z* @23.32** | 135,793,265 | 21,341,128 | 75,125,460 | 97,906,617 | 103,923,101 | 4,761,112 | 64,784,992 | 93,779,129 | 67,154,626 | 113,992,411 | 91,519,814 | 75,261,397 | 216,762,918 |
| **329.23 *m/z* @23.72** | 6,140,166 | 13,397,131 | 74,849,350 | 4,379,248 | 24,030,707 | 4,060,740 | 26,772,971 | 10,673,592 | 7,014,179 | 9,605,490 | 7,752,189 | 71,955,021 | 16,791,685 |
| **301.20 *m/z* @24.33** | 16,142,333 | 4,542,611 | 7,799,398 | 11,117,526 | 6,535,148 | 1,646,544 | 2,175,227 | 10,289,654 | 7,378,487 | 12,665,576 | 10,415,972 | 3,347,872 | 5,265,630 |
| **327.22 *m/z* @24.92** | - | - | - | - | - | - | - | - | - | - | - | - | 64,072,291 |
| **287.22 *m/z* @24.94** | 16,584,743 | 4,904,901 | 6,803,378 | 8,346,236 | 5,386,984 | 1,563,727 | 3,007,528 | 14,693,080 | 9,350,263 | 12,846,526 | 9,580,351 | 3,294,267 | 11,035,746 |
| **229.14 *m/z* @25.05** | 7,303,524 | 10,365,462 | 10,673,879 | 3,851,226 | 8,883,271 | 3,102,469 | 9,434,766 | 4,470,739 | 12,908,886 | 12,588,822 | 11,497,503 | 5,852,581 | 6,808,045 |
| **187.13 *m/z* @25.74** | 12,104,760 | 20,514,505 | 25,169,891 | 7,011,333 | 16,230,090 | 5,431,649 | 19,466,183 | 8,688,297 | 30,407,483 | 25,036,571 | 20,638,118 | 9,805,254 | 11,219,993 |
| **426.97 *m/z* @25.96** | 10,400,944 | - | - | - | - | - | - | - | 7,730,780 | - | - | - | - |
| **601.15 *m/z* @26.14** | - | - | - | - | - | - | - | - | - | - | - | - | 47,229,714 |
| **311.17 *m/z* @27.12** | 62,910,453 | 66,448,669 | 65,174,751 | 55,203,205 | 51,705,927 | 46,937,638 | 49,063,077 | 42,820,245 | 47,609,876 | 45,677,487 | 59,730,138 | 61,732,781 | 55,239,174 |
| **247.10 *m/z* @27.17** | 13,437,902 | 2,188,571 | 12,219,700 | 3,292,905 | 8,238,893 | 513,232 | 15,683,403 | 3,131,184 | 3,983,889 | 47,165,110 | 32,983,343 | 14,500,688 | 23,019,172 |
| **255.07 *m/z* @27.39** | 28,999,479 | 3,243,495 | 20,645,976 | 24,628,160 | 27,167,725 | 667,440 | 18,818,906 | 21,953,551 | 14,916,183 | 29,971,969 | 23,991,090 | 17,071,294 | 94,490,865 |
| **339.20 *m/z* @27.54** | 70,930,922 | 41,182,221 | 29,276,133 | 31,612,962 | 39,410,758 | 44,591,073 | 55,887,980 | 61,246,881 | 65,323,064 | 63,009,332 | 70,358,133 | 104,671,571 | 101,685,362 |
| **325.18 *m/z* @27.64** | 744,685,108 | 684,450,422 | 668,084,945 | 621,437,393 | 526,334,413 | 468,953,999 | 415,239,122 | 359,961,654 | 266,541,658 | 228,885,157 | 200,902,617 | 171,774,875 | 128,788,327 |
| **263.13 *m/z* @27.85** | - | - | - | - | - | - | - | - | - | 433,275 | 413,740 | - | 45,668,102 |

- denotes not detectable; values highlighted in orange are Manuka-specific peaks. All *m/z* values are [M-H]^-^. For clarity, only average areas are shown but SE was < 3 %.

**Table. S3. Correlation of peak abundance with antimicrobial activity against different bacteria**

RED = more inhibitory, BLUE = less inhibitory. As noted in the text, certain compounds are often correlated as effective against the same sets of bacteria – e.g. *Klebsiella* and *E. faecalis.*

Please note that the first four bacteria were tested against all honey samples whereas the second set (*Klebsiella*, *E. coli* and *S. aureus*) were only tested against a reduced set of honeys. Therefore, the two separate correlations are shown. However, the components suggested to be antimicrobial (in purple) are consistent across the 2 correlations.

The criteria for the selection of AM components is arbitrary and was based on a R value of >0.33 in more than 5 bacteria. The selection of components in blue which appeared to “help growth” was based on a R value < -0.50 against 5 bacteria.

| ***m/z* & RT** | **MRSA** | ***E. faecalis*** | ***A. baumannii*** | ***P. aeruginosa*** |  | ***K. pneumoniae*** | ***E. coli*** | ***S. aureus*** |
| --- | --- | --- | --- | --- | --- | --- | --- | --- |
| 581.17 *m/z* *@* RT 12.64 | 0.30 | 0.20 | 0.29 | 0.25 |  | 0.37 | 0.61 | 0.29 |
| 290.12 *m/z* *@* RT 12.70 | 0.36 | 0.35 | 0.41 | 0.26 |  | 0.52 | 0.64 | 0.48 |
| 165.06 *m/z* *@* RT 12.49 | 0.00 | 0.06 | 0.21 | -0.15 |  | 0.00 | 0.10 | -0.42 |
| 177.02 *m/z* *@* RT 12.73 | 0.01 | 0.06 | 0.21 | -0.15 |  | -0.01 | 0.11 | -0.41 |
| 153.02 *m/z* *@* RT 12.84 | -0.21 | 0.11 | 0.01 | -0.33 |  | 0.05 | 0.07 | -0.57 |
| 236.09 *m/z* *@* RT 13.15 | 0.01 | 0.04 | 0.30 | -0.05 |  | 0.30 | 0.12 | -0.36 |
| 285.13 *m/z* *@* RT 13.23 | -0.76 | -0.09 | -0.91 | -0.86 |  | -0.93 | -0.59 | -0.82 |
| 365.18 *m/z* *@* RT 13.43 | 0.05 | 0.12 | 0.41 | 0.21 |  | 0.56 | 0.14 | 0.05 |
| 151.04 *m/z* *@* RT 13.59 | 0.41 | 0.45 | 0.49 | 0.31 |  | 0.66 | 0.64 | 0.67 |
| 569.21 *m/z* *@* RT 13.62 | -0.15 | -0.03 | 0.29 | -0.02 |  | 0.34 | -0.07 | -0.29 |
| 225.11 *m/z* *@* RT 13.90 | -0.71 | -0.08 | -0.67 | -0.76 |  | -0.61 | -0.55 | -0.90 |
| 317.03 *m/z* *@* RT 14.01 | 0.02 | -0.09 | 0.28 | 0.01 |  | 0.19 | 0.00 | -0.38 |
| 419.12 *m/z* *@* RT 14.13 | 0.05 | -0.12 | -0.03 | 0.29 |  | 0.36 | 0.12 | 0.57 |
| 173.08 *m/z* *@* RT 14.38 | -0.01 | 0.15 | 0.35 | 0.01 |  | 0.38 | 0.27 | -0.16 |
| 377.18 *m/z* *@* RT 14.46 | 0.04 | 0.27 | 0.16 | 0.00 |  | 0.58 | 0.24 | 0.15 |
| 165.05 *m/z* *@* RT 14.66 | -0.19 | 0.05 | 0.19 | -0.16 |  | 0.21 | -0.11 | -0.48 |
| 227.13 *m/z* *@* RT 14.68 | -0.60 | -0.09 | -0.83 | -0.56 |  | -0.68 | -0.63 | -0.38 |
| 639.15 *m/z* *@* RT 14.87 | -0.59 | 0.00 | -0.54 | -0.58 |  | -0.48 | -0.50 | -0.88 |
| 203.13 *m/z* *@* RT 14.98 | 0.20 | 0.00 | 0.51 | 0.36 |  | 0.62 | 0.34 | 0.11 |
| 163.04 *m/z* *@* RT 15.06 | 0.41 | 0.35 | 0.39 | 0.28 |  | 0.56 | 0.74 | 0.49 |
| 361.15 *m/z* *@* RT 15.17 | -0.15 | -0.05 | 0.27 | -0.03 |  | 0.30 | -0.05 | -0.34 |
| 369.19 *m/z* *@* RT 15.28 | -0.29 | -0.19 | -0.17 | -0.21 |  | -0.37 | -0.53 | -0.71 |
| 919.23 *m/z* *@* RT 15.63 | 0.01 | -0.12 | -0.03 | 0.24 |  | -0.74 | -0.52 | -0.47 |
| 523.20 *m/z* *@* RT 15.66 | -0.36 | 0.04 | 0.06 | -0.28 |  | 0.12 | -0.18 | -0.52 |
| 361.15 *m/z* *@* RT 15.69 | -0.12 | -0.05 | 0.30 | 0.01 |  | 0.33 | -0.02 | -0.27 |
| 361.15 *m/z* *@* RT 16.13 | -0.22 | -0.07 | 0.24 | -0.11 |  | 0.24 | -0.08 | -0.42 |
| 363.17 *m/z* *@* RT 16.38 | -0.11 | -0.07 | 0.28 | 0.03 |  | 0.33 | 0.00 | -0.27 |
| 361.15 *m/z* *@* RT 16.52 | -0.14 | -0.07 | 0.28 | 0.00 |  | 0.32 | -0.02 | -0.31 |
| 363.17 *m/z* *@* RT 16.77 | -0.15 | -0.13 | 0.27 | 0.01 |  | 0.29 | -0.06 | -0.26 |
| 347.17 *m/z* *@* RT 16.84 | -0.20 | -0.17 | 0.01 | -0.06 |  | 0.12 | 0.15 | -0.45 |
| 363.17 *m/z* *@* RT 17.16 | -0.15 | -0.08 | 0.25 | -0.03 |  | 0.29 | -0.05 | -0.34 |
| 187.10 *m/z* *@* RT 17.23 | 0.04 | 0.34 | 0.06 | -0.15 |  | 0.19 | 0.32 | -0.14 |
| 349.19 *m/z* *@* RT 17.44 | -0.12 | -0.15 | 0.11 | 0.07 |  | 0.25 | 0.15 | -0.38 |
| 187.10 *m/z* *@* RT 17.66 | -0.07 | 0.06 | 0.01 | -0.17 |  | 0.08 | 0.20 | -0.26 |
| 144.05 *m/z* *@* RT 17.73 | 0.35 | 0.43 | 0.40 | 0.31 |  | 0.71 | 0.39 | 0.77 |
| 345.15 *m/z* *@* RT 17.88 | -0.12 | -0.34 | 0.09 | 0.09 |  | -0.15 | -0.19 | -0.42 |
| 273.08 *m/z* *@* RT 18.17 | -0.18 | -0.23 | -0.02 | -0.09 |  | 0.01 | -0.38 | -0.31 |
| 347.17 *m/z* *@* RT 18.53 | -0.40 | -0.26 | -0.28 | -0.23 |  | -0.53 | -0.41 | -0.65 |
| 849.36 *m/z* *@* RT 18.68 | -0.44 | -0.16 | -0.34 | -0.41 |  | -0.37 | -0.35 | -0.90 |
| 273.08 *m/z* *@* RT 18.86 | -0.74 | -0.11 | -0.94 | -0.83 |  | -0.92 | -0.55 | -0.65 |
| 309.13 *m/z* *@* RT 18.98 | 0.35 | 0.27 | 0.33 | 0.30 |  | 0.52 | 0.65 | 0.49 |
| 263.13 *m/z* *@* RT 19.00 | 0.35 | 0.27 | 0.33 | 0.30 |  | 0.53 | 0.66 | 0.50 |
| 199.10 *m/z* *@* RT 19.24 | -0.01 | -0.05 | 0.29 | 0.12 |  | 0.32 | 0.20 | -0.15 |
| 343.80 *m/z* *@* RT 19.75 | 0.34 | 0.29 | 0.58 | 0.23 |  | 0.62 | 0.58 | 0.04 |
| 263.13 *m/z* *@* RT 19.94 | 0.23 | 0.17 | 0.22 | 0.25 |  | 0.50 | 0.50 | 0.46 |
| 201.11 *m/z* *@* RT 20.11 | 0.01 | -0.09 | 0.30 | 0.15 |  | 0.35 | 0.22 | -0.11 |
| 349.19 *m/z* *@* RT 20.57 | -0.46 | -0.28 | -0.34 | -0.22 |  | -0.39 | -0.36 | -0.62 |
| 687.31 *m/z* *@* RT 20.59 | -0.21 | 0.01 | -0.11 | -0.22 |  | -0.46 | -0.35 | -0.37 |
| 463.25 *m/z* *@* RT 20.76 | -0.46 | -0.08 | -0.72 | -0.59 |  | -0.70 | -0.25 | -0.52 |
| 325.18 *m/z* *@* RT 21.05 | 0.41 | 0.35 | 0.40 | 0.27 |  | 0.55 | 0.75 | 0.51 |
| 395.19 *m/z* *@* RT 21.06 | -0.20 | -0.54 | 0.09 | 0.10 |  | 0.02 | -0.24 | -0.49 |
| 582.26 *m/z* *@* RT 21.28 | -0.51 | -0.33 | -0.39 | -0.49 |  | -0.54 | -0.45 | -0.90 |
| 285.80 *m/z* *@* RT 21.33 | -0.22 | 0.06 | 0.02 | -0.28 |  | 0.02 | 0.11 | -0.57 |
| 582.26 *m/z* *@* RT 21.68 | -0.35 | -0.24 | -0.38 | -0.43 |  | -0.49 | -0.27 | -0.70 |
| 628.27 *m/z* *@* RT 21.94 | -0.09 | 0.17 | -0.19 | -0.31 |  | -0.24 | 0.05 | -0.26 |
| 628.27 *m/z* *@* RT 22.43 | -0.15 | 0.24 | -0.01 | -0.25 |  | -0.05 | 0.13 | -0.45 |
| 327.22 *m/z* *@* RT 22.51 | 0.21 | 0.12 | 0.31 | 0.06 |  | 0.28 | 0.26 | -0.16 |
| 271.06 *m/z* *@* RT 23.32 | -0.32 | -0.05 | -0.50 | -0.52 |  | -0.58 | 0.12 | -0.54 |
| 329.23 *m/z* *@* RT 23.72 | 0.21 | 0.12 | 0.29 | 0.06 |  | 0.29 | 0.26 | -0.12 |
| 301.20 *m/z* *@* RT 24.33 | -0.50 | 0.10 | -0.60 | -0.63 |  | -0.57 | -0.04 | -0.57 |
| 287.22 *m/z* *@* RT 24.94 | -0.46 | -0.06 | -0.52 | -0.45 |  | -0.51 | -0.04 | -0.47 |
| 229.14 *m/z* *@* RT 25.05 | 0.11 | -0.14 | 0.29 | 0.25 |  | 0.36 | 0.38 | 0.05 |
| 187.13 *m/z* *@* RT 25.74 | 0.12 | -0.21 | 0.31 | 0.31 |  | 0.40 | 0.32 | -0.05 |
| 426.97 *m/z* *@* RT 25.96 | -0.64 | -0.15 | -0.62 | -0.48 |  | -0.74 | -0.52 | -0.47 |
| 311.17 *m/z* *@* RT 27.12 | -0.35 | 0.30 | -0.28 | -0.53 |  | -0.07 | -0.15 | -0.25 |
| 247.10 *m/z* *@* RT 27.17 | 0.28 | 0.12 | 0.30 | 0.14 |  | 0.32 | 0.72 | 0.20 |
| 255.07 *m/z* *@* RT 27.39 | -0.19 | -0.11 | -0.34 | -0.39 |  | -0.48 | 0.21 | -0.53 |
| 339.20 *m/z* *@* RT 27.54 | 0.40 | 0.10 | 0.08 | 0.30 |  | 0.15 | 0.54 | 0.45 |
| 325.18 *m/z* *@* RT 27.64 | -0.82 | -0.11 | -0.58 | -0.77 |  | -0.60 | -0.91 | -0.67 |

Components in purple and blue are putatively identified in Table 2A and 2B respectively in the manuscript.

As an example, the component with *m/z* 247 (C_14_H_15_O_4_) only showed a positive correlation with antimicrobial effects against *E. coli*. This formula and MS^2^ data matches with a component often found in propolis (Fabris et al., 2013) and identified in honeys as dimethylallyl caffeate (Lori et al., 2019).

Another isomer of ABA was detected in the honey samples, but it was not correlated with antimicrobial activity. The order of elution suggests that first ABA peak may be *trans-*abscisic acid (which more readily formed FA adducts) whilst the second was *cis*-abscisic acid (Oelschlaegel et al., 2012; Fyfe et al., 2017).

The presence of tri-*p*-coumaroyl spermidine has been noted previously in honeys (Negri et al., 2018) and this component was present as a cluster of 3 peaks, which had different amounts of FA adducts noted at *m/z* [M-H]^-^ = 628. Only two of these peaks was detected as pro-microbial possibly due to the splitting of the signal with higher amounts of the FA adduct. On further inspection, there was also a peak at *m/z* 785 which eluted slightly later, which gave an exact mass formula of C_46_H_50_N_4_O_8_ for the [M-H]^-^ ion, which matches with tetra-coumaroyl spermine also found in honeys (Folly, 2019). This component also gave MS^2^ data consistent with losses of spermine and coumaroyl groups.

References are given in the manuscript except:

Fabris et al. (2013) Antioxidant Properties and Chemical Composition Relationship of European and Brazilian Propolis. Pharmacology & Pharmacy, 4, 46-51.

Folly (2019). The impact of plant chemicals on bee health: Interactions with parasites and immunity. Royal Holloway University, PhD thesis.

Lori et al. (2019). Honey extracts inhibit PTP1B, upregulate insulin receptor expression and enhance glucose uptake in human HepG2 cells. Biomed Pharmacother, 113: 108752.

|  |  |  |  |  |  |  |  |  |  |  |  |  |  |  |  |  |  |  |  |  |  |  |  |  |  |  |  |  |  |  |  |  |  |  |  |
| --- | --- | --- | --- | --- | --- | --- | --- | --- | --- | --- | --- | --- | --- | --- | --- | --- | --- | --- | --- | --- | --- | --- | --- | --- | --- | --- | --- | --- | --- | --- | --- | --- | --- | --- | --- |

|  |  |  |  |  |  |  |  |  |  |  |  |  |  |  |  |  |  |  |  |  |  |  |  |  |  |  |  |  |  |  |  |  |  |  |  |
| --- | --- | --- | --- | --- | --- | --- | --- | --- | --- | --- | --- | --- | --- | --- | --- | --- | --- | --- | --- | --- | --- | --- | --- | --- | --- | --- | --- | --- | --- | --- | --- | --- | --- | --- | --- |
